# [^225^Ac]Ac-labeled matuzumab is an effective radioimmunotherapeutic against EGFR-positive triple negative breast cancer

**DOI:** 10.1186/s13058-026-02220-z

**Published:** 2026-01-16

**Authors:** Anjong Florence Tikum, Dede Api Fon, Fabrice Ngoh Njotu, Nikita Henning, Emmanuel Nwangele, Hanan Babeker, Jessica Pougoue Ketchemen, Alireza Doroudi, Maruti Uppalapati, Humphrey Fonge

**Affiliations:** 1 Department of Medical Imaging, College of Medicine, University of Saskatchewan, Saskatoon, SK S7N 0W8 Canada; 2 Faculté de Pharmacie, Université Laval, Ferdinand Vandry Pavillon, Québec, QC G1V 0A6 Canada; 3 Axe Oncologie, Centre de Recherche du CHU de Québec-Université Laval, Québec, QC G1J 5B3 Canada; 4 Department of Pathology and Laboratory Medicine, College of Medicine, University of Saskatchewan, Saskatoon, SK S7N 5E5 Canada

**Keywords:** EGFR, Radioimmunotherapy, TNBC, Alpha particle therapy, Actinium-225

## Abstract

**Background:**

EGFR is overexpressed in TNBC, and “naked” anti-EGFR monoclonal antibodies have been evaluated in clinical trials with dismal effectiveness. Matuzumab is an anti-EGFR monoclonal antibody that can be used to develop theranostics. We posit that compared with “naked” antibodies, [^225^Ac]Ac-Macropa-matuzumab will be effective against EGFR-positive TNBC xenografts.

**Methods:**

We developed and characterized [^225^Ac]Ac-Macropa-matuzumab. Cytotoxicity was studied in EGFR-positive MDA-MB-468 (high EGFR density), MDA-MB-231 (medium EGFR density) and MCF-7 (low EGFR density) 2D monolayer cells and 3D spheroids using live-cell imaging. Biodistribution was carried out in naïve female BALB/c and athymic nude BALB/c tumor-bearing mice. Radioimmunotherapy was studied after administration of 2 × 13 kBq [^225^Ac]Ac-Macropa-matuzumab dose and compared with irrelevant IgG and saline-treated controls. Safety was evaluated in naïve female BALB/c mice.

**Results:**

Biodistribution of [^225^Ac]Ac-Macropa-matuzumab in mice bearing MDA-MB-468 and MDA-MB-231 xenografts showed the highest tumor uptake at 120 h post-injection (p.i.) and was 48.3 $\:\pm\:$ 28.6%IA/g and 39.0 $\:\pm\:\:$ 9.1%IA/g, respectively. In vitro, [^225^Ac]Ac-Macropa-matuzumab suppressed the growth of EGFR-positive spheroids with an IC_50_ of: MDA-MB-468 (5.3 $\:\pm\:$ 6.6 kBq/mL) ∼ MDA-MB-231 (4.9 $\:\pm\:$ 6.4 kBq/mL) < MCF-7 (132.7 $\:\pm\:$ 42.6 kBq/mL). [^225^Ac]Ac-Macropa-matuzumab demonstrated favourable biodistribution and was cleared from most non-target organs by day-10 p.i. 57% of mice bearing MDA-MB-468 xenograft treated with [^225^Ac]Ac-Macropa-matuzumab had complete remission (CR). Less pronounced effect was observed for MDA-MB-231 xenograft.

**Conclusion:**

[^225^Ac]Ac-Macropa-matuzumab was safe and effective against EGFR-positive TNBC.

**Supplementary Information:**

The online version contains supplementary material available at 10.1186/s13058-026-02220-z.

## Introduction

Breast cancer (BC) is the most commonly diagnosed cancer in women [1]. Triple negative breast cancer (TNBC) is a subtype of BC, that is characterized by the absence of the expression of estrogen receptor (ER), progesterone receptor (PgR), and human epidermal growth factor receptor 2 (HER2). TNBC accounts for 15–20% of all BCs and it is linked to high early onset, high rate of relapse and relatively short survival rate upon metastasis [2]. The standard of care for TNBC is dependent on disease stage and primarily consists of surgical resection followed by chemotherapy [3]. Taxanes and/or anthracyclines constitute the first line of treatment for metastatic BC with response rates of 38 and 46% for single agent and combination-based therapies, respectively [4]. However, *de novo* resistance to taxanes is common in > 50% of patients and most patients acquire resistance [4]. A few druggable cell surface targets including vascular endothelial growth factor receptor II (VEGFR2), fibroblast growth factor receptor (FGFR), glycoprotein non-metastatic B (GPNMB), trophoblast antigen 2 (TROP-2) and epidermal growth factor receptor (EGFR) have been preclinically and clinically investigated against TNBC [5–8]. Of these, anti-TROP2 antibody-drug conjugate (ADC) sacituzumab govitecan (SG) is the only cell-surface targeted therapeutics approved against TNBC [9].

The ErbB/HER family is composed of four growth factor receptors namely EGFR (erbB1 or HER1), erbB2 (HER2; or neu in rodents), erbB3 (HER3), and erbB4 (HER4) that share a high degree of sequence homology. Signaling through EGFR, HER2, and HER3 promotes tumor cell proliferation and survival in a variety of epithelial malignancies notably in metastatic breast cancer (MBC) [10]. EGFR is overexpressed in all breast cancer subtypes, but more so in TNBC and inflammatory breast cancer (IBC) [11–13], making it an ideal target for treatment of the disease. Two classes of targeted molecules have been used to target EGFR in BC patients; tyrosine kinase inhibitors (TKIs) and monoclonal antibodies. TKIs as monotherapy or in combination with chemotherapy has shown no benefits in this population with at best overall response rates (ORR) of 0–9% [14–16]. Anti-EGFR monoclonal antibodies such as cetuximab have shown modest benefits in patients in combination with other agents [17–19]. Of all the clinical studies using anti-EGFR antibodies in TNBC the best response was with the addition of cetuximab to cisplatin with an ORR of 20% (vs. 10% for cisplatin alone). This study also showed a modest increase in overall survival of 3 months vs. 1.5 months [17, 20]. Evidently, naked antibodies and TKIs have not led to significant improvements for EGFR-positive TNBC and none of these are approved.

Radioimmunotherapy (RIT) is a targeted form of cancer treatment that combines radiation therapy with the specificity of antibodies/fragments to deliver potent radiation to cancer cells [21]. It has gained a lot of clinical interests, particularly, alpha emitting RIT (αRIT) due to durable responses observed in patients with various solid organ malignancies [22–29]. α-emitters have the highest linear energy transfer (LET) among therapeutic radioisotopes due to their high energy (5–8 MeV) and short path length in tissue (50–80 μm) which makes them superior to Auger electron-emitters and beta-emitters [30]. The high LET of ionizing radiation causes irreparable double-stranded DNA breaks which further leads to cell death [31, 32]. A short path length confines cytotoxic effects to a small area (2–5 cell layers) around the decay site [30]. This makes alpha particles perfect for targeting cancer cells while reducing toxicity to healthy tissues. Furthermore, they are effective even in low-oxygen environments where other treatments often fail [33].

Matuzumab is a humanized monoclonal antibody of the immunoglobin IgG1-subclass that binds selectively to EGFR and inhibits ligand-mediated activation [34]. Other authors have evaluated anti-EGFR antibodies radiolabeled with beta ([^177^Lu]Lu and [^90^Y]Y) or Auger-electron emitters ([^111^In]In) preclinically with very modest effects at controlling tumor growth [35–37]. With the exception of Solomon et al. [38] published previously by our group there are no other studies of alpha-emitting anti-EGFR radioimmunoconjugates against EGFR-positive TNBC. For the first time, we have radiolabeled matuzumab with alpha-emitting actinium-225 ([^225^Ac]Ac) and evaluated its cytotoxicity in vitro and in vivo effectiveness against mouse models of TNBC.

## Materials and methods

### Cells and reagents

All reagents and solvents obtained from commercial suppliers were used without any further purification. TNBC cells with high (MDA-MB-468, RRID: CVCL_0419), medium (MDA-MB-231, RRID: CVCL_0062) and low (MCF-7, RRID: CVCL_0031) EGFR densities [39] were used for in vitro and in vivo studies. The cells were cultured in Dulbecco’s Modified Eagle Medium (DMEM) (Hyclone Laboratories, Logan, UT). All cultured media used were supplemented with 10% FBS (Biochrom, Sigma-Aldrich, St Louis, MO) and 1% penicillin/streptomycin (Hyclone Laboratories, Logan, UT). Matuzumab (RRID: AB_3695392) was obtained from Icorbio (Wayne PA) and was buffered exchanged to DPBS prior to use. Bifunctional chelator 6-((16-((6-carboxypyridin-2-yl)methyl)-1,4,10,13-tetraoxa-7,16-diazacyclooctadecan-7-yl)methyl)-4-isothiocyanatopicolinic acid (*p*-SCN-Macropa) was obtained from Ratio Therapeutics (Boston, MA) under a material transfer agreement. Accelerator-produced [^225^Ac]Ac-nitrate (> 99% purity) containing trace amounts of [^227^Ac]Ac (0.6%) was purchased from Oak Ridge National Laboratory (Oak Ridge, TN).

### Conjugation and quality control

We conjugated eighteen-membered macrocyclic ring bifunctional chelator *p*-SCN-Macropa to matuzumab for radiolabeling with [^225^Ac]Ac as previously reported [40]. To do this, a stock of 20 mg/mL of Macropa-SCN was prepared for conjugation reactions. Matuzumab was buffer exchanged to 0.1 M sodium bicarbonate buffer, pH 9, containing 0.15 M sodium chloride using Amicon Ultra-4 30 K centrifugal filters (EMD Millipore, Burlington, MA, USA. After buffer exchange, a 15-mole excess of the chelator was added to the antibody solution and incubated at 4 °C, shaking at 750 rpm for 18 h. After incubation, the conjugated antibody was then purified by buffer exchanging into PBS to remove excess unreacted chelator. The purity of immunoconjugates was determined using size exclusion chromatography, high-performance liquid chromatography (SEC-HPLC). Briefly, for SEC-HPLC analysis, ~ 40 µg of sample in 100 µL of PBS was injected and allowed to run in PBS as mobile phase for 30 min at a flowrate of 0.45 mL/min using a Waters 2796 Separations Module and a Waters 2487 Dual λ Absorbance Detector, mounted with XBride Protein BEH SEC Column, 200 A, 3.5 μm, 7.8 mm × 150 mm (Cat. 186007639, Waters Corporation). The UV detector was fixed at 254 nm and 280 nm. Mass spectrometry (MS) of matuzumab and Macropa-matuzumab was done using matrix-assisted laser desorption ionization-time of flight (MALDI-TOF) at the Alberta Proteomics and Mass Spectrometry Facility, University of Alberta (Edmonton, AB), and was used to calculate the chelator to antibody ratio. Briefly, 1 μL of each sample was mixed with 1 μL of sinapinic acid (10 mg/mL in 50% acetonitrile/water + 0.1% trifluoroacetic acid). One μL of the sample/matrix solution was then spotted onto a stainless-steel target plate and allowed to air dry. Mass spectra were obtained using an UltrafleXtreme MALDI-TOF/TOF instrument (Bruker Daltonic GmbH). All MS spectra were recorded in positive linear mode and external calibration was performed by use of a standard protein mixture. Furthermore, the size and purity were characterized using electronic electrophoresis (2100 bioanalyzer system, Santa Clara CA) using Agilent High Sensitivity Protein 250 Kit (cat # 5067-1575) following the manufacturer’s protocol. The size and relative peak area were calculated using Agilent 2100 Expert software.

### Radiolabeling and stability of radioimmunoconjugate

To radiolabel the immunoconjugate with actinium-225, [^225^Ac]Ac-nitrate was added to a 1.5 mL microtube, and the activity was determined using a dose calibrator. To this, 150 mM ammonium acetate (pH 6.0), L-ascorbic acid, and Macropa-matuzumab were added. The reaction pH was checked by applying 1 µL of the mixture onto Hydrion pH paper (5.0–9.0 range, Sigma-Aldrich), with a typical value around 5.8. The mixture was then incubated at 37 °C with shaking at 650 rpm for 2 h. Following incubation, 1–5 µL of the sample was analyzed on an instant thin-layer chromatography (iTLC) strip (silica gel-impregnated paper iTLC-SG, Agilent Technologies, Santa Clara, CA) using 50 mM sodium citrate buffer (pH 5.2) as mobile phase to assess [^225^Ac]Ac incorporation into the antibody. The purification of [^225^Ac]Ac-labeled tracer was conducted using Amicon Ultra-4 centrifugal filters (10 K, EMD Millipore, Burlington, MA, USA) with PBS. The purity of the radiolabeled immunoconjugates was determined using size exclusion radio-HPLC and iTLC. A radiochemical purity > 95% was usually considered sufficient for in vitro and in vivo experiments.

The in vitro stability of the radioimmunoconjugate was assessed in PBS and human serum. [^225^Ac]Ac-Macropa-matuzumab was incubated in PBS and human serum at 37 °C, 300 rpm for a total of 12 days. During the 12-day incubation period, 10 µL of the mixture was spotted on an iTLC strip using 50 mM sodium citrate buffer (pH 5.2) as mobile phase. Stability was assessed on days 0, 2, 3, 7, 10, and 12 by reading the iTLC strips in a gamma counter (Hidex AMG, serial # 2240351) and calculating the percentage of actinium-225 still incorporated into the antibody.

### Flow cytometry and internalization

Flow cytometry analysis was performed using BD Accuri™ C6 Plus flow cytometer (BD Bioscience, Franklin Lakes NJ) to determine the binding of Macropa-matuzumab to EGFR-positive MDA-MB-468 or MDA-MB-231 cells in comparison with matuzumab [41]. A 9–11-point curve allowed for the determination of K_D_ and EC_50_ of the antibodies. Briefly, in a 96-well non-binding plate, different concentrations (1000–0.006nM) of matuzumab and Macropa-matuzumab were added to MDA-MB-468 or MDA-MB-231 cells seeded at of 5 × 10^5^ cells per well and left to incubate at 4 °C for 30 min. Cells were washed with cold 1X DPBS. Goat anti-Human IgG PE-conjugated secondary antibody (eBioscience, cat. #12-4998-82) was added to cells (1 in 100 dilution) and allowed to bind for at least 30 min. Cells were then washed twice, resuspended in 1X DPBS, and read using the BD Accuri™ C6 Plus flow cytometer. Experiments were carried out in triplicate. The mean fluorescence intensity was converted to percentage bound and plotted against concentration, and a nonlinear curve fitting was used to estimate the K_D_ and EC_50_. The data were analyzed using FlowJo v10 (BD Biosciences, Ashland OR) and GraphPad Prism 9.

To evaluate internalization in MDA-MB-468, MDA-MB-231 and MCF-7 cell lines, the cells were seeded in flat-bottom 96-well plates and incubated for 48 h before matuzumab was added. IncuCyte^®^ FabFluor reagent (Essen BioScience, Ann Arbor, MI) was conjugated with the antibody at a molar ratio of 3:1 in complete growth media for 15 min at 37 °C. Media containing FabFluor-conjugated antibody were then added to the cells in triplicate prior to imaging using IncuCyte^®^ S3 live-cell imager (Essen BioScience, Ann Arbor, MI) [42]. The area under the curve (AUC (µm^2^h)) was determined from the total red object area versus time curve using GraphPad Prism Version 10 (Boston, MA, USA).

### 2D and 3D In vitro cytotoxicity

The in vitro cytotoxicity of matuzumab and [^225^Ac]Ac-Macropa-matuzumab was determined using an IncuCyte S3 Live cell imaging system (Essen BioScience, Ann Arbor, MI, USA) in MDA-MB-468, MDA-MB-231, and MCF-7 cell lines [43]. Briefly, cells were seeded in a 96-well flat-bottom Corning plate pre-coated with poly-D-lysine (10000 cells per well) 24 h before treatment. The cells were washed with 1X PBS treated with different concentrations of either 2000–0.033 nM of matuzumab or 250–0.48 KBq/mL of [^225^Ac]Ac-Macropa-matuzumab in growth media containing Incucyte^®^ Cytotox Red reagent and incubated at 37 °C for 30 min before imaging. Live-cell images were captured every 2 h using a 10× objective lens using phase contrast and fluorescence channel. During each scanning, 4 images were acquired until the end of the experiment. All cell images were processed and analyzed using Incucyte S3 software. The relative fluorescent values generated were used to calculate the IC_50_ values using GraphPad Prism 10.

For 3D spheroids, after harvesting the cells (MDA-MB-468, MDA-MB-231 and MCF-7) and counting, cells for spheroid growth (5000–10000) were seeded in a PrimeSurface 96-well U-bottom plate (MS-9096UZ) at a final volume of 100 μL. The plate was then centrifuged at 319.47 RCF for 10 min and placed in an incubator (day 0). On day 1, 100 μL of medium containing 6 μg/mL of collagen 1 was added to each well for a final collagen 1 concentration of 3 μg/mL. The plate was centrifuged at 319.47 RCF for 10 min and placed in an incubator. On day 3, 100 μL of medium was pipetted out, and the spheroids were then treated with different concentrations (2000–3.9 nM) of matuzumab or 250–0.48 kBq/mL of [^225^Ac]Ac-Macropa-matuzumab in media containing Incucyte® Cytotox Red reagent. The plate was again centrifuged at 319.47 RCF for 10 min and incubated at 37 °C for 30 min before imaging. Spheroid size and growth inhibition were determined using IncuCyte S3 Live-cell imaging system. Live-cell images were captured every 6 h using a 10× objective lens using phase contrast and fluorescence channel. All spheroid images were processed and analyzed using IncuCyte S3 software. The fluorescent red count generated was converted to percentage inhibition and plotted against concentration to determine the IC_50_ values using GraphPad Prism v10 (RRID: SCR_002798).

### Safety in naïve mice

Safety studies were conducted using six-week-old female BALB/c mice (RRID: IMSR_CRL:028) (*n* = 4 per group) obtained from Charles Rivers. Acute (Day 2) toxicity was studied after a 13 kBq single dose injection of [^225^Ac]Ac-Macropa-matuzumab. A second group of mice was administered 13 kBq [^225^Ac]Ac-Macropa-matuzumab at day-0 and day-10 and sacrificed at day-14. Blood was collected via cardiac puncture and samples were analyzed for CBC and clinical chemistry at the Prairie Diagnostic Service at the Western College of Veterinary Medicine (University of Saskatchewan, Saskatoon SK). The results were analyzed and plotted using GraphPad Prism v10 (RRID: SCR_002798).

### Ex vivo biodistribution and dosimetry

All animal studies were approved by the University of Saskatchewan Animal Care and Use Committee following the guidelines outlined in the Use of Laboratory Animals (protocol # 20220021). Female athymic nude BALB/c mice were obtained from Charles River (St-Constant, QC) at 4 weeks of age and housed in a 12 h light, 12 h dark cycle in a temperature and humidity-controlled vivarium. Animals had *ad libitum* access to diet (Lab Diet, St. Louis, MI) and water [43]. After one week of acclimatization, mice were subcutaneously injected with a suspension of 5 × 10^6^ EGFR-positive MDA-MB-468 or 10 × 10^6^ MDA-MB-231 breast cancer cells in 100 µL of a 1:1 mixture of PBS and Matrigel matrix basement membrane (Discovery Laboware, Inc. Bedford MA) at the hind limb of each mouse. Tumor growth was monitored by measuring the length and width of each tumor using a digital caliper. Tumor volume was calculated using the formula: volume = ½(length × width^2^).

Ex vivo biodistribution of [^225^Ac]Ac-Macropa-matuzumab was studied in tumor-bearing athymic BALB/c nude (RRID: IMSR_CRL:490) (*n* = 3 or 4 per group) and healthy BALB/c mice (*n* = 4 per group). Tumor-bearing athymic BALB/c nude mice (MDA-MB-468 xenograft on left hind leg and MDA-MB-231 xenograft on right hind leg with average body weight of 20.5 g) or healthy BALB/c mice (average weight = 18.7 g) were injected with 13 kBq of [^225^Ac]Ac-Macropa-matuzumab via a tail vein. The mice were euthanized at different time points (5, 24, 72, 120 and 240 h) post-injection, and the carcasses were collected and analyzed using a gamma counter (Wallac Wizard 1470 PerkinElmer, Waltham MA) using the full range of [^225^Ac]Ac gamma energies (100–440.5 KeV). The activity was expressed as injected activity (%IA) and %IA per gram organ weight (%IA/g). The mouse biodistribution (%IA/g) data was extrapolated to human data (%IA) using the formula % IA (human) = %IA/g (mouse) × total body weight of mouse (in kg) × mass of (female) human organ (in g) / total body weight of (female) human (in kg). For each organ, this was plotted against sampling time and used to obtain an estimate of the residence time of the agent in the organ in MBq-h/MBq, represented by the area under the time-activity function integrated to infinity (complete decay) of the [^225^Ac]Ac. The residence time was fitted into the OLINDA kinetics model using the International Commission for Radiological Protection (ICRP) Adult Female model (OLINDA/EXM V2.2, Hermes Medical Solutions Montreal QC) to generate absorbed doses in units of mGy/MBq of [^225^Ac]Ac administered. Bone marrow dose was estimated from whole bone and cross dose to the organ.

### Radioimmunotherapy

When MDA-MB-231 or MDA-MB-468 xenograft had reached an average volume of 100 ± 50 mm^3^ or 50 $\:\pm\:\:25\:$mm^3^, respectively, mice were randomized into 3 different groups (n $\:\ge\:$ 6 per group) and each injected via a tail vein with either saline, matuzumab (15 mg/kg) on days 0, 6 and 11 or 2 × 13 kBq of [^225^Ac]Ac-Macropa-matuzumab on days 0 and 10. The study was terminated when tumor volume reached $\:\ge\:$ 1500 mm^3^, and this was used to determine survival in the different groups using Kaplan-Meier curves. Anti-tumor effect was expressed as % tumor growth inhibition (%TGI) of test groups relative to control groups using the formula (1-∆T/∆C) × 100, where ∆T and ∆C are the differences between final and initial tumor volumes of test and control groups, respectively.

### Statistical analyses

All data were expressed as the mean ± standard error of mean of at least three independent experiments. A two-tailed Student’s t-test or analysis of variance (ANOVA) with Bonferroni post hoc test was used to assess the statistical significance between the groups. All graphs were prepared and analyzed using GraphPad Prism (v10; GraphPad, La Jolla, CA, USA) and a *p*-value < 0.05 was considered significant.

## Results

### Quality control, internalization and radiochemistry

**Fig. 1:**
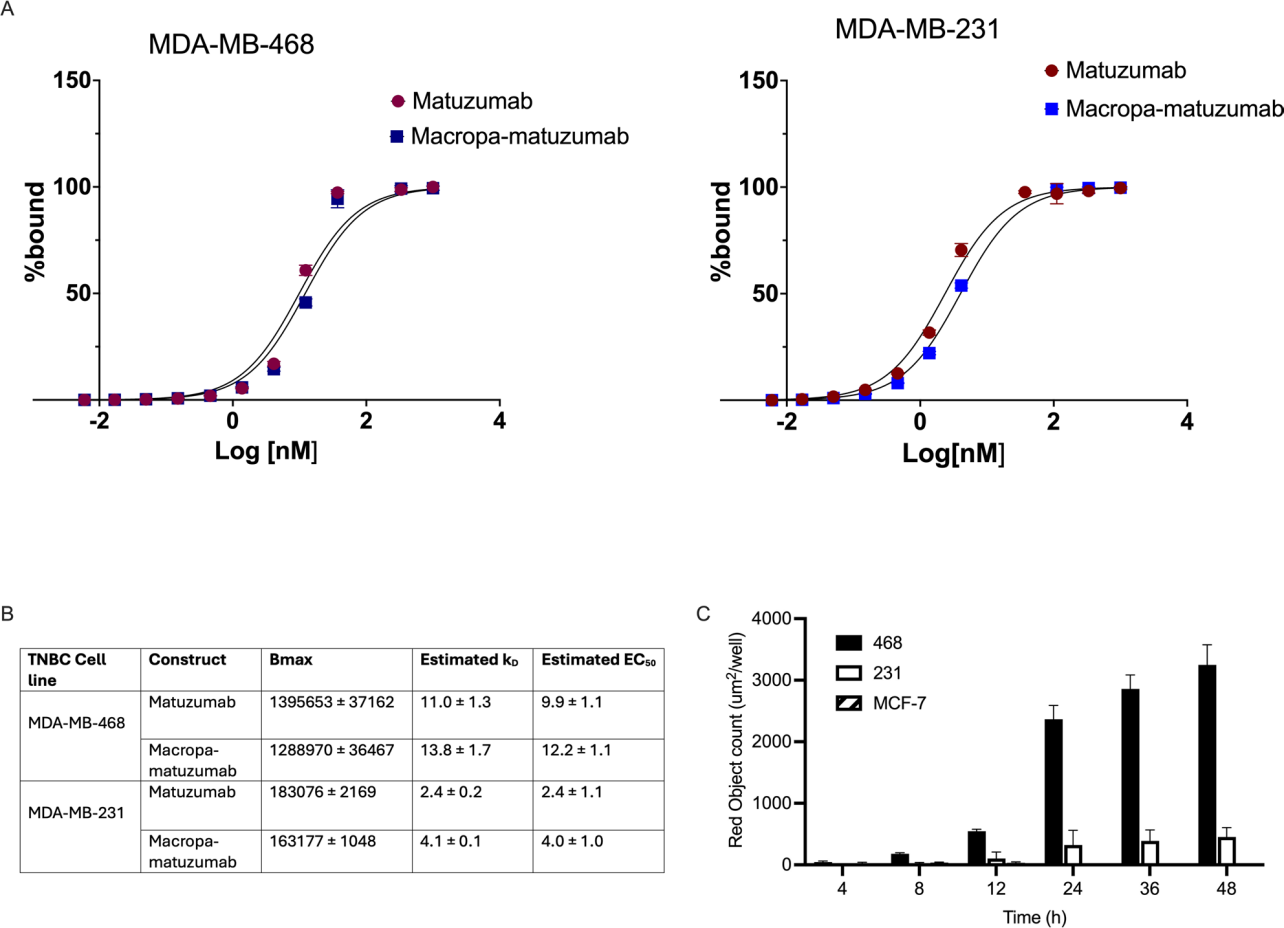
In vitro flow cytometry analysis of matuzumab and Macropa-matuzumab in EGFR-positive MDA-MB-468 (high EGFR density) and MDA-MB-231 (medium EGFR density) cells. **A** The mean fluorescence intensity was converted to percentage bound and plotted against concentration, and a nonlinear curve fitting was used to estimate the EC_50_ and the K_D_. **B** A summary table of the estimated Bmax, K_D_ and EC_50_ for matuzumab and Macropa-matuzumab in different EGFR-positive cell lines. **C** Internalization of matuzumab in different EGFR-positive cell lines

The conjugation of Macropa-matuzumab was obtained as a clear solution with no milky appearance. The immunoconjugate was characterized for size and binding to EGFR using bioanalyzer and flow cytometry. Bioanalyzer indicated that the molecular weight of matuzumab was 155.5 ± 4.5 kDa, while that of Macropa-matuzumab was 157 ± 1.0 kDa (Supplementary Figure S1A and B). The SEC-HPLC purity of Macropa-matuzumab was ≥ 98% with no aggregates (Supplementary Figure S1C). MALDI-TOF result showed a chelator to antibody ratio of 4.5 (Supplementary Figure S1D). Flow cytometry showed that matuzumab effectively bound to EGFR-positive MDA-MB-468 and MDA-MB-231. There were no significant differences in the dissociation constant (K_D_) and EC_50_ for matuzumab and Macropa-matuzumab in MDA-MB-468 and MDA-MB-231 cell lines, indicating that conjugation did not affect the binding to EGFR (Fig. 1A and B, Supplementary Figure S2).

The ability of matuzumab to internalize into the cells was obtained by calculating the area under the internalization curve (total red count) at 0–48 h post-treatment. In all cell lines tested (MDA-MB-468, MDA-MB-231 and MCF-7), internalization was highest at 48 h post-treatment and was dependent on the expression of EGFR density on cells (Fig. 1C). At 24 h post-treatment, the internalization of matuzumab in MDA-MB-468 (high expression 2366.9 $\:\pm\:$ 223.7 µm^2^/well) was 7 times greater than MDA-MB-231 (medium expression 322.6 $\:\pm\:$ 238.6 µm^2^/well), and 556.1 times greater than in MCF-7 (low expression, 4.3 $\:\pm\:$ 7.4 µm^2^/well).

Macropa-matuzumab was efficiently radiolabeled using [^225^Ac]Ac, yielding a high radiochemical yield of > 95% and a radiochemical purity of > 99% (after purification using an Amicon 30 K filter). The efficiency of labeling and purity was confirmed using radio-SEC-HPLC and radio-iTLC (Supplementary Figure S1E). The radiochemical stability of [^225^Ac]Ac-Macropa-matuzumab was investigated using iTLC at 37 °C in PBS and human serum. The stability of [^225^Ac]Ac-Macropa-matuzumab was > 65% and > 38% after 10 days in human serum and PBS, respectively (Supplementary Figure S3).

### In vitro cytotoxicity

Live-cell imaging was used to study the cytotoxicity of matuzumab and [^225^Ac]Ac-Macropa-matuzumab. As expected, [^225^Ac]Ac-Macropa-matuzumab was more potent than matuzumab in all cell lines. In MDA-MB-468 cell line, IC_50_ of [^225^Ac]Ac-Macropa-matuzumab (1.9 $\:\pm\:$ 1.0 nM) was 35-fold lower than that of matuzumab (65.3 $\:\pm\:$ 54.0 nM). Similar trend was observed for MDA-MB-231 cells (Table 1 and Supplementary Figure S4). Matuzumab had no effect on MCF-7 but enhanced cytotoxicity was observed for [^225^Ac]Ac-Macropa-matuzumab (8.9 $\:\pm\:\:$8.1 nM).

**Table 1:** IC_50_ of [^225^Ac]Ac-Macropa-matuzumab in TNBC cell lines

	MDA-MB-468	MDA-MB-231	MCF-7
Matuzumab (nM)	65.3 $\:\pm\:$ 53.9	50.1 $\:\pm\:$ 12.1	-
[^225^Ac]Ac-Macropa-matuzumab (kBq/mL)	2.8 $\:\pm\:$ 0.5	6.6 $\:\pm\:$ 12.0	13.3 $\:\pm\:$ 6.2
[^225^Ac]Ac-Macropa-matuzumab (nM)	1.9 $\:\pm\:$ 0.3	4.4 $\:\pm\:$ 7.9	8.8 $\:\pm\:$ 4.1

In vitro cytotoxicity of matuzumab and [^225^Ac]Ac-Macropa-matuzumab was further studied in MDA-MB-468, MDA-MB-231 and MCF-7 breast cancer spheroids (Table 2 and Supplementary Figure S5). In MDA-MB-468, the IC_50_ of [^225^Ac]Ac-Macropa-matuzumab (3.6 $\:\pm\:$ 4.3 nM) was 17.5-fold lower than that of matuzumab (62.4 $\:\pm\:$ 9.3 nM). A similar trend was observed for MDA-MB-231 (100.3 $\:\pm\:$ 90.51 nM compared with 3.22 $\:\pm\:$ 4.2 nM for matuzumab and [^225^Ac]Ac-Macropa-matuzumab, respectively). Matuzumab had no effect on MCF-7 spheroid. However, enhanced cytotoxicity was observed for [^225^Ac]Ac-Macropa-matuzumab (88.34 $\:\pm\:$ 27.9 nM).

**Table 2:** IC_50_ of [^225^Ac]Ac-Macropa-matuzumab in TNBC spheroids

	MDA-MB-468	MDA-MB-231	MCF-7
Matuzumab (nM)	62.4 $\:\pm\:$ 9.3	100.3 $\:\pm\:$ 90.5	–
[^225^Ac]Ac-Macropa-matuzumab (kBq/mL)	5.3 $\:\pm\:$ 6.6	4.9 $\:\pm\:$ 6.4	132.7 $\:\pm\:$ 42.6
[^225^Ac]Ac-Macropa-matuzumab (nM)	3.6 $\:\pm\:$ 4.3	3.2 $\:\pm\:$ 4.2	88.3 $\:\pm\:$ 27.9

### Safety in naïve mice

**Fig. 2:**
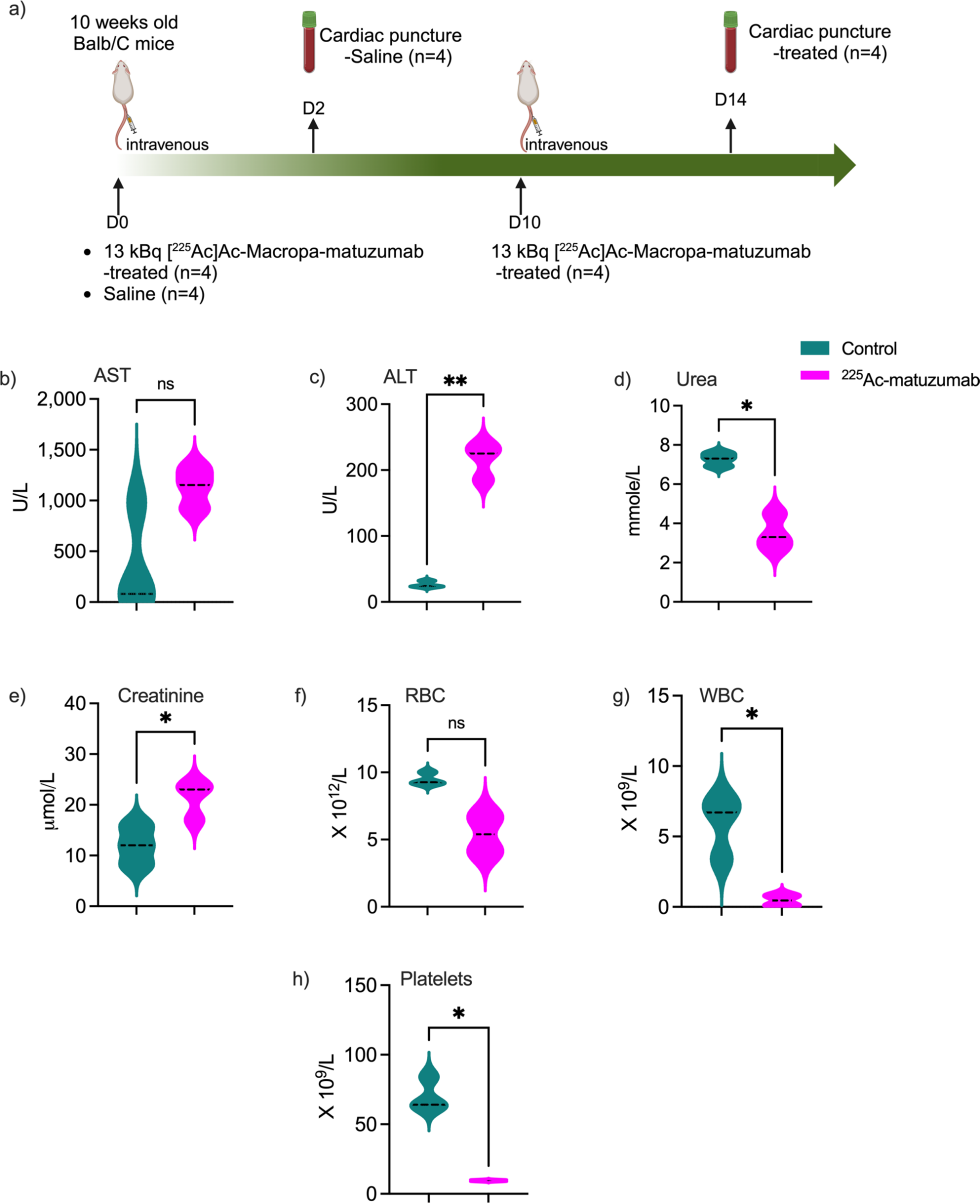
Normal tissue toxicity of [^225^Ac]Ac-Macropa-matuzumab in female naïve BALB/c mice. **A** Experimental design. Mice were administered 2 × 13 kBq [^225^Ac]Ac-Macropa-matuzumab via a tail vein and sacrificed 14 days after the second dose for CBC and clinical chemistry analyses, **B** aspartate aminotransferase (AST), **C** alanine aminotransferase (ALT), **D** Urea, **E** Creatinine, **F** red blood cell count (RBC), **G** white blood cell count (WBC), **H** Platelets

Normal tissue toxicity of [^225^Ac]Ac-Macropa-matuzumab was evaluated in healthy female BALB/c mice after one or two doses of 13 kBq administered at 10 days apart. [^225^Ac]Ac-Macropa-matuzumab seemed tolerated by female mice although some significant differences were observed in white blood cell (WBC), mean corpuscular hemoglobin concentration (MCHC), platelets, urea, creatinine, alanine transferase (ALT), aspartate aminotransferase (AST), glutamate dehydrogenase (GLDH) counts (Fig. 2 and Supplementary Table 1). However, no mouse in the treatment groups experienced > 20% decrease in bodyweight. Mice bearing MDA-MB-468 tumors and treated with [^225^Ac]Ac-Macropa-matuzumab had an average bodyweight of 25.50 ± 0.45 g on day 0 and 25.70 ± 0.60 g on day 58. Similarly, mice bearing MDA-MB-231 tumors had a mean bodyweight of 24.17 ± 1.05 g on day 0 and 23.40 ± 1.60 g on day 58. The average bodyweight of the control groups ranged between 22.57 ± 0.30 to 24.83 ± 0.47 g (MDA-MB-468) and between 25.60 ± 0.51 to 28.50 g (MDA-MB-231) from start to endpoint.

### Ex vivo biodistribution and dosimetry

**Fig. 3:**
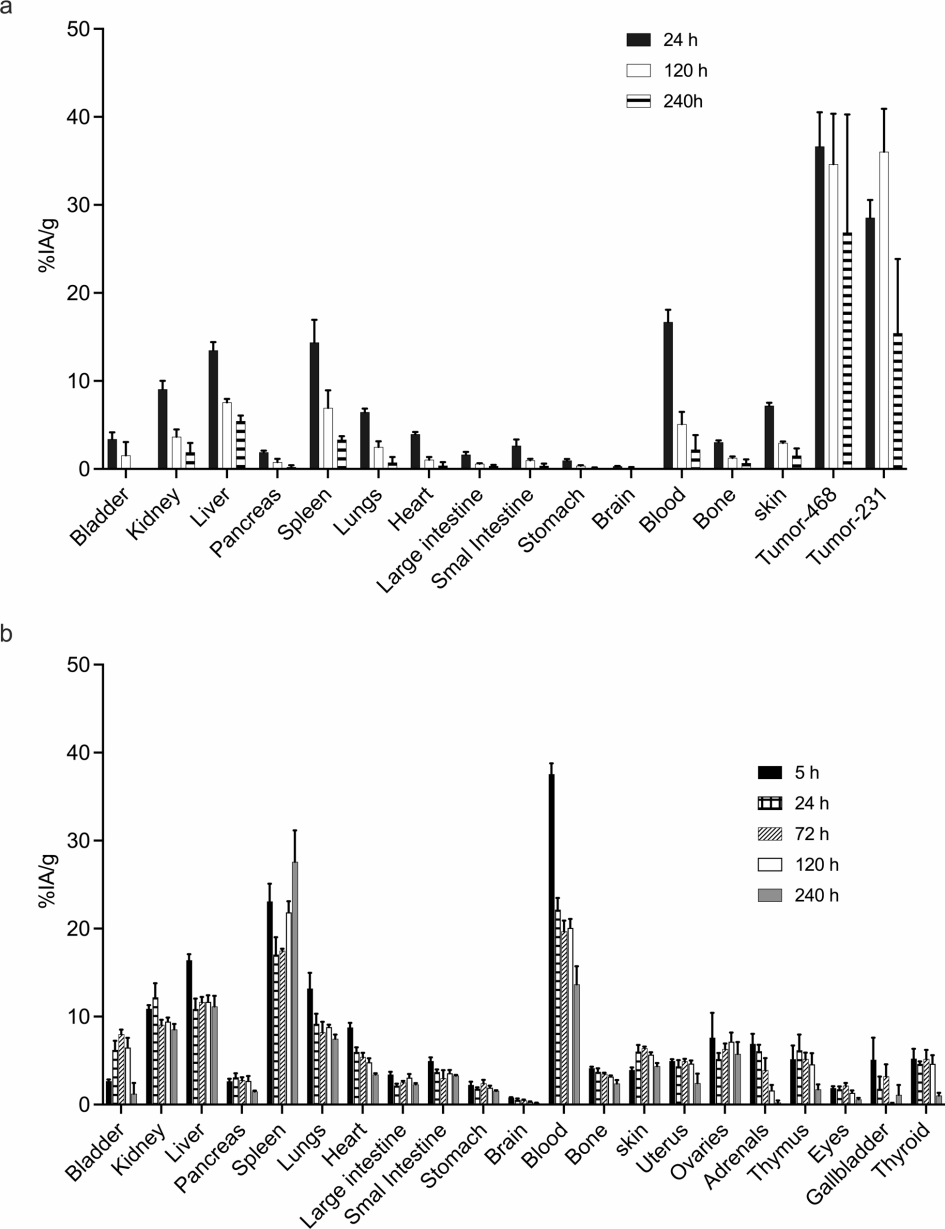
Biodistribution of [^225^Ac]Ac-Macropa-matuzumab in tumor bearing and naïve mice. **A** Biodistribution in BALB/c athymic nude mice (*n* = 3 per time point) bearing both MDA-MB-468 (on left flank) and MDA-MB-231 (on right flank) xenografts. **B** Biodistribution in naïve BALB/c mice (*n* = 4 per time point) expressed as % injected activity per gram (%IA/g). Mice were administered 13 kBq [^225^Ac]Ac-Macropa-matuzumab via a tail vein and sacrificed at different time points. Activity in the organs was counted using a gamma counter (Wallac Wizard 1470 PerkinElmer, Waltham MA) using the full range of [^225^Ac]Ac gamma energies (100–440.5 KeV)

Biodistribution of [^225^Ac]Ac-Macropa-matuzumab was evaluated in both athymic nude tumor-bearing BALB/c and healthy BALB/c mice (Fig. 3). Athymic nude tumor-bearing mice (MDA-MB-468 and MDA-MB-231) or healthy BALB/c were injected with 13 kBq of [^225^Ac]Ac-Macropa-matuzumab. The mice were sacrificed at different time points, and the carcasses were collected and measured using a gamma counter. In healthy mice, uptake was high in the liver, spleen, kidney and blood at early time points, but only the spleen (27.6 $\:\pm\:$ 6.16%IA/g) and blood (13.6 $\:\pm\:$ 3.57%IA/g) had high uptake at 10 days post-injection (Supplementary Table 2). In the tumor-bearing mice, uptake of [^225^Ac]Ac-Macropa-matuzumab was higher in the tumor than other organs at all time points, with the highest uptake at 120 h (MDA-MB-468 tumor, 34.61 $\:\pm\:$ 9.93%IA/g; MDA-MB-231 tumor, 36.03 $\:\pm\:\:$8.44%IA/g; liver, 7.54 $\:\pm\:\:$0.68%IA/g; spleen, 6.92 $\:\pm\:\:$3.47%IA/g; kidney, 3.64 $\:\pm\:$ 1.42%IA/g; blood, 5.07 $\:\pm\:$ 2.40%IA/g) (Supplementary Table 3).

Uptake and elimination of [^225^Ac]Ac-Macropa-matuzumab from mouse tissues was used to project the radiation absorbed doses using OLINDA kinetics model in human female adults (Table 3). Human organ dosimetry calculations showed the organs receiving the highest dose are the spleen > liver > lungs.

**Table 3:** Human radiation dose estimates (female) of [^225^Ac]Ac-Macropa-matuzumab

Organs	mGy/MBq female
Adrenals	2.18E + 02
Brain	2.09E + 01
Breast	2.79E + 01
Esophagus	9.69E-01
Eyes	1.03E + 02
Gallbladder	1.24E−00
Left colon	4.59E−01
Small intestine	3.70E−00
Stomach	8.39E−01
Right colon	1.56E−00
Rectum	1.49E−01
Heart wall	3.88E + 02
Kidneys	6.98E + 02
Liver	1.05E + 03
Lungs	7.60E + 02
Ovaries	4.41E + 02
Pancreas	1.59E + 02
Salivary glands	7.89E−02
Red marrow	3.52E−01
Spleen	1.68E + 03
Thymus	2.35E + 02
Thyroid	4.48E + 02
Bladder	1.07E-01
Uterus	1.50E + 02
Total body	4.71E + 01
Effective dose	1.94E + 02

### Radioimmunotherapy

**Fig. 4:**
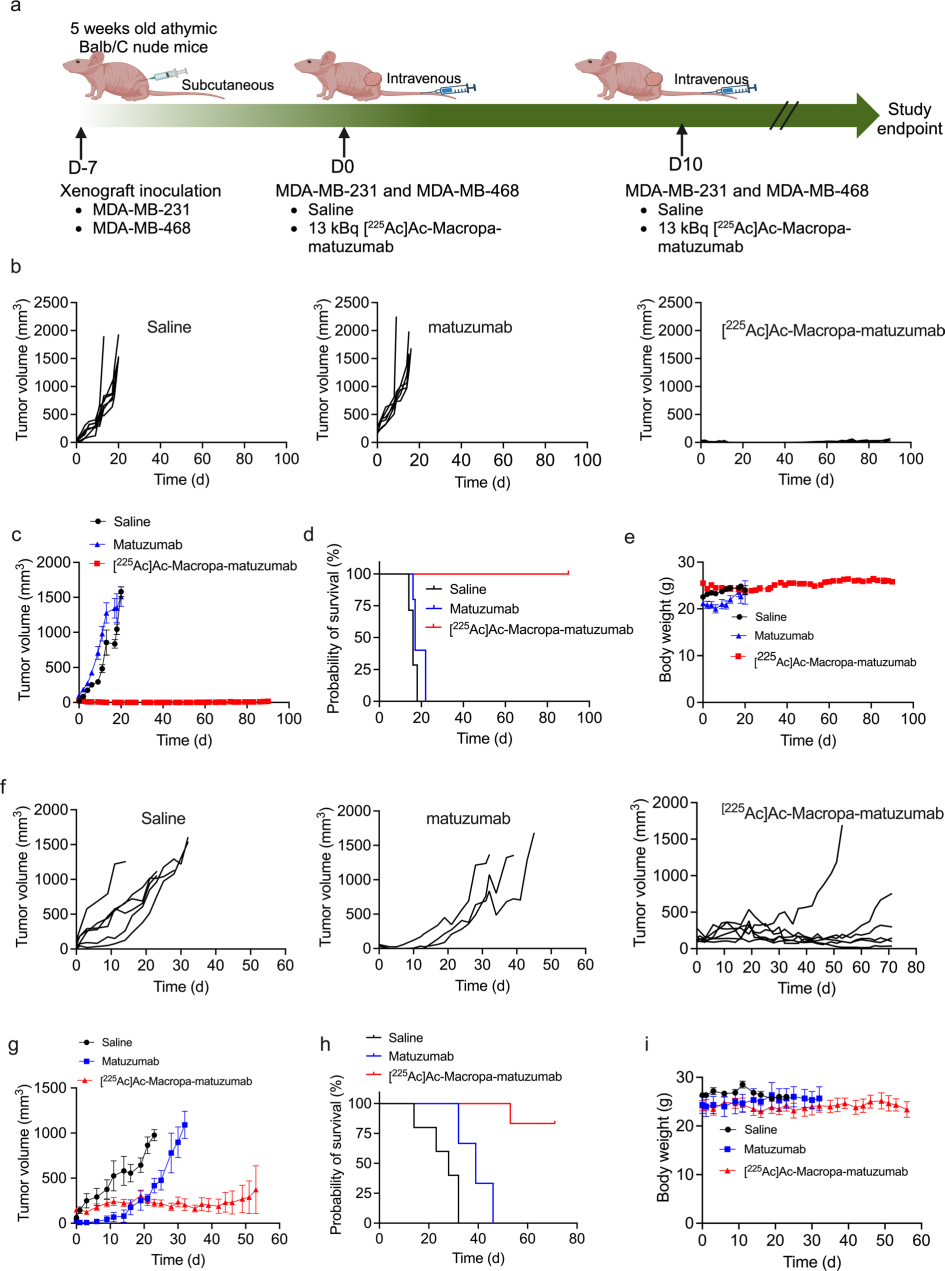
Therapeutic effectiveness of [^225^Ac]Ac-Macropa-matuzumab in EGFR-positive MDA-MB-468 (high EGFR density) and MDA-MB-231 (medium EGFR density) xenografts. **A** Experimental design, **B** Individual tumor volume plots for saline, matuzumab and [^225^Ac]Ac-Macropa-matuzumab for MDA-MB-468 xenograft, **C** Average tumor volumes plot for MDA-MB-468 xenograft, **D** Kaplan survival curve of MDA-MB-468 xenograft, **E** Plots of body weights for different groups, **F** Individual tumor volume plot of saline, matuzumab and [^225^Ac]Ac-Macropa-matuzumab for MDA-MB-231 xenograft, **G** Average tumor volume plot for MDA-MB-231 xenograft, **H** Kaplan survival curve of MDA-MB-231 xenograft, and, **I** Plot of body weights for different groups

The efficacy of [^225^Ac]Ac-Macropa-matuzumab was evaluated in EGFR-positive TNBC xenografts MDA-MB-468 and MDA-MB-231. Mice were treated with either saline, 3 doses of 15 mg/kg of matuzumab on days 0, 6, and 11 or 2 doses of 13 kBq of [^225^Ac]Ac-Macropa-matuzumab 10 days apart (n $\:\ge\:$ 6 per group) (Fig. 4A). For MDA-MB-468, mice treated with [^225^Ac]Ac-Macropa-matuzumab had complete remission by day 20. However, three out of ten mice experienced regrowth but did not reach endpoint by the end of the study (average volume of 53 ± 18.21 mm^3^ at end of study). The median survival did not differ significantly between the saline group and the matuzumab-treated group, with both groups having a median survival of 16 or 17 days, respectively. However, the median survival was not reached with [^225^Ac]Ac-Macropa-matuzumab treatment (Fig. 4B, C and D). The tumor growth inhibition, expressed as a percentage (%TGI_max_) at 20 days post treatment, was 8% for matuzumab and 101.5% for [^225^Ac]Ac-Macropa-matuzumab. [^225^Ac]Ac-Macropa-matuzumab was also effective in controlling the growth of MDA-MB-231 xenograft, although no complete remissions were observed. The median survival was 28 and 39 days for saline and matuzumab, respectively. However, the median survival was not reached for the [^225^Ac]Ac-Macropa-matuzumab treatment (Fig. 4F, G and H). %TGI_max_ was 58.3% for matuzumab and 92% for [^225^Ac]Ac-Macropa-matuzumab against MDA-MB-231.

## Discussion

EGFR is an attractive target for TNBC because it is overexpressed in most patients. EGFR-targeted TKIs and monoclonal “naked” antibody therapeutics have been evaluated in many clinical trials against TNBC with very underwhelming results [17–19, 44, 45]. Cetuximab and panitumumab are anti-EGFR monoclonal antibodies that are approved for the treatment of KRAS wild-type colorectal cancer and have been evaluated in many clinical trials against TNBC. Yardley et al. reported the result of a phase II trial adding panitumumab to gemcitabine, and carboplatin in patients with metastatic TNBC [46]. Progression-free survival of 4.4 months of the combination was less than historic clinical outcome of gemcitabine, and carboplatin in this population [47, 48]. Similarly, the addition of cetuximab to cisplatin compared with cisplatin against metastatic TNBC failed to meet clinical endpoint [17]. Matuzumab has not been evaluated in clinical trials against TNBC. Given the poor outcomes observed with the use of unconjugated monoclonal antibodies this study was undertaken to evaluate the effectiveness of anti-EGFR antibody matuzumab radiolabeled with potent alpha particle emitter [^225^Ac]Ac.

A few studies have evaluated the effectiveness of anti-EGFR monoclonal antibodies radiolabeled with β^−^ and Auger electron emitting radionuclides. However, most preclinical and clinical data show αRIT is more effective than βRIT and Auger electron RIT [49, 50]. αRIT is up to 1000-fold more potent than βRIT due to their high LET. High LET leads to more ionisation events along the decay track and therefore higher rates of double strand breaks per decay, which translates into higher biological effectiveness [49, 51, 52], β^−^RITs suffer from two dose-limiting drawbacks: (1) the long range of β^−^ photon is toxic to adjacent healthy tissues, and (2) its low LET. We conjugated matuzumab with eighteen-membered macrocyclic bifunctional chelator SCN-Macropa. Prior to radiolabeling with [^225^Ac]Ac, Macropa-matuzumab was characterized for binding, purity and internalization in EGFR-positive MDA-MB-468, MDA-MB-231 and MCF-7 cells. As expected, internalization of the immunoconjugate was dependent on EGFR density. Macropa-matuzumab was radiolabeled with [^225^Ac]Ac resulting in a stable complex. The stability of [^225^Ac]-Ac-Macropa-matuzumab was determined at different time points at 37 °C in PBS and human plasma using iTLC. More than 96% of [^225^Ac]Ac-Macropa-matuzumab remained intact for 120 h in human serum and was 91% stable in PBS confirming earlier reports on the stability of Macropa as a bifunctional chelator for [^225^Ac]Ac [40].

3D spheroids are a more relevant in vitro model than monolayer cells when studying cytotoxicity, particularly because they more accurately mimic micrometastatic lesions for which αRIT are most effective. The effectiveness of matuzumab vs. [^225^Ac]Ac-Macropa-matuzumab were evaluated using 3D spheroids grown using MDA-MB-468, MDA-MB-231 and MCF-7 cells. In all spheroids tested, [^225^Ac]Ac-Macropa-matuzumab was 17- to 31-fold more potent than the unlabeled matuzumab including in low EGFR-expressing MCF-7 for which matuzumab showed no activity. This indicates that [^225^Ac]Ac-Macropa-matuzumab can be effective in patients with varying levels of EGFR expression as well as in lesions with heterogenous antigen expression.

We evaluated the safety of 2 × 13 kBq [^225^Ac]Ac-Macropa-matuzumab (after 14 days of 2nd dose) in naïve BALB/c mice and observed significant differences in WBC, MCHC, platelets, urea, creatinine, ALT, AST, and GLDH which are indicative of sub-acute kidney, liver and hemopoietic toxicities. Despite this, no gross evidence in toxicity such as weight loss was observed for [^225^Ac]Ac-Macropa-matuzumab treated mice. Since delayed (> 3 months) toxicity has been observed with [^225^Ac]Ac-labeled compounds, a more extensive delayed safety study would be required for the agent. Treatment of mice bearing MDA-MB-468 xenografts using 2 × 13 kBq [^225^Ac]Ac-Macropa-matuzumab resulted in complete remission in 4/7 mice while the remaining 3/7 mice had an average tumor volume of 53 mm^3^ at the end of the study. Untreated and matuzumab treated mice reached 1500 mm^3^ by day 16. Our group previously evaluated [^89^Zr]Zr-DFO-nimotuzumab-SpyTag-∆N-SpyCatcher and [^225^Ac]Ac-Macropa-nimotuzumab-SpyTag-∆N-SpyCatcher as theranostic in MDA-MB-468 model [38]. The highest tumor uptake of [^89^Zr]Zr-DFO-nimotuzumab-SpyTag-∆N-SpyCatcher was 6.0%IA/g which was significantly lower than the 48.4%IA/g obtained with [^225^Ac]Ac-Macropa-matuzumab in this study. Consequently, treatment of mice bearing MDA-MB-468 xenograft with 2 × 16.6 kBq [^225^Ac]Ac-Macropa-nimotuzumab-SpyTag-∆N-SpyCatcher was only minimally effective [38]. A few authors have evaluated Auger-electron emitting and β^−^ emitting radioimmunotherapeutics in EGFR-positive TNBC models with less exciting effectiveness as monotherapy [35–38]. Facca et al. evaluated anti-EGFR Auger-electron emitting [^111^In]In-DOTA-panitumumab in NOD-SCID mice bearing MDA-MB-231 xenograft [35]. The highest average tumor uptake of [^111^In]In-DOTA-panitumumab in this model was 18%IA/g compared with 39.0%IA/g obtained for [^225^Ac]Ac-Macropa-matuzumab in athymic nude BALB/c mice bearing MDA-MB-231 xenograft in this study. Treatment of NOD-SCID mice bearing MDA-MB-231 with 22 MBq had far less pronounced effects compared with [^225^Ac]Ac-Macropa-matuzumab. Monotherapy using [^177^Lu]Lu-labeled anti-EGFR antibody (derived from cetuximab) was less effective against athymic BALB/c nude mice bearing MDA-MB-231 xenograft, however triple therapy using the radiopharmaceutical with chemotherapy (docetaxel and doxorubicin) and a poly(adenosine diphosphate ribose) polymerase (PARP) inhibitor [36] resulted in complete remission in 93.3% of mice with no tumor regrowth.

## Conclusion

We have developed an actinium-225-labeled anti-EGFR antibody and evaluated the [^225^Ac]Ac-Macropa-matuzumab against EGFR-positive TNBC for the first time. [^225^Ac]Ac-Macropa-matuzumab showed remarkable effectiveness against EGFR-positive TNBC models. Given the dismal effectiveness of naked anti-EGFR monoclonal antibodies and TKIs in TNBC patients, and the lack of durable responses of Auger-electron emitting and β^−^-emitting radioimmunoconjugates targeting this antigen preclinically, this positive data deserves further evaluation in additional animal models and potentially in a phase 1 trial.

## Supplementary Information

Below is the link to the electronic supplementary material.

Supplementary Material 1

## Data Availability

All data generated or analysed during this study are included in this published article (and its supplementary information files).

## Abbreviations

ADC: Antibody-drug conjugate
ALP: Alkaline phosphatase
ALT: Alanine transaminase
AST: Aspartate aminotransferase
BC: Breast cancer
BFC: Bifunctional chelators
CBC: Complete blood count
CR: Complete remission
DFO: p-isothiocyanatobenzyl desferrioxamine
DOTA: 1,4,7,10-tetraazacyclododecane-1,4,7,10-tetracetic acid
DPBS: Dulbecco’s phosphate-buffered saline
EC_50_: Half maximal effective concentration
EGFR: Epidermal growth factor receptor I
FBS: Fetal bovine serum
HER2: Human epidermal growth factor receptor II
HPLC: High Performance Liquid Chromatography
HS: Human serum
iTLC: Instant thin layer chromatography
Macropa: (6-((16-((6-carboxypyridin-2-yl)methyl)-1,4,10,13-tetraoxa-7,16-diazacyclooctadecan-7-yl)methyl)-4-isothiocyanatopicolinic acid
MBC: Metastatic breast cancer
MS: Mass spectrometry
PBS: Phosphate buffered saline
PET: Positron emission tomography
p.i.: Post injection
RBC: Red blood cells
RDW: Red blood cell distribution width
SDS-PAGE: Sodium dodecyl-sulfate polyacrylamide gel electrophoresis
STR: Short tandem repeat
TAT: Targeted alpha therapy
TGI: Tumor growth inhibition
TKI: Tyrosine kinase inhibitors
TNBC: Triple negative breast cancer
WBC: White blood cells
