# Supplementary material for: [225Ac]Ac-labeled matuzumab is an effective radioimmunotherapeutic against EGFR-positive triple negative breast cancer
Source: Breast Cancer Res. 2026 Jan 16;28:44. doi: 10.1186/s13058-026-02220-z (PMC12892558; doi:10.1186/s13058-026-02220-z)
Supplement: Supplementary file 1 — Supplementary Material 1 [file 13058_2026_2220_MOESM1_ESM.docx]

**[^225^Ac]Ac-labeled matuzumab is an effective radioimmunotherapeutic against EGFR-positive triple negative breast cancer**

*Anjong Florence Tikum^a^, Dede Api Fon^b,c^, Fabrice Ngoh Njotu^b,c^, Nikita Henning^a^, Emmanuel Nwangele^,b,c^, Hanan Babeker^a^, Jessica Pougoue Ketchemen^b,c^, Alireza Doroudi^a^, Maruti Uppalapati^d^, Humphrey Fonge^a,b,c *^*

^a^Department of Medical Imaging, University of Saskatchewan, College of Medicine, Saskatoon SK, S7N 0W8, Canada

^b^Faculté de Pharmacie, Université Laval, Ferdinand Vandry Pavillon, Québec, QC G1V 0A6, Canada

^c^Axe Oncologie, Centre de Recherche du CHU de Québec-Université Laval, Québec, QC G1J 5B3, Canada

^d^Department of Pathology and Laboratory Medicine, University of Saskatchewan, College of Medicine, Saskatoon SK, S7N 5E5, Canada

***Corresponding Author**:

Humphrey Fonge, PhD

Axe Oncologie, Centre de recherche du CHU de Québec-Université Laval, Québec, QC G1J 5B3, Canada

E-mail: humphrey.fonge@crchudequebec.ulaval.ca

ORCID ID : 0000-0001-9388-6872


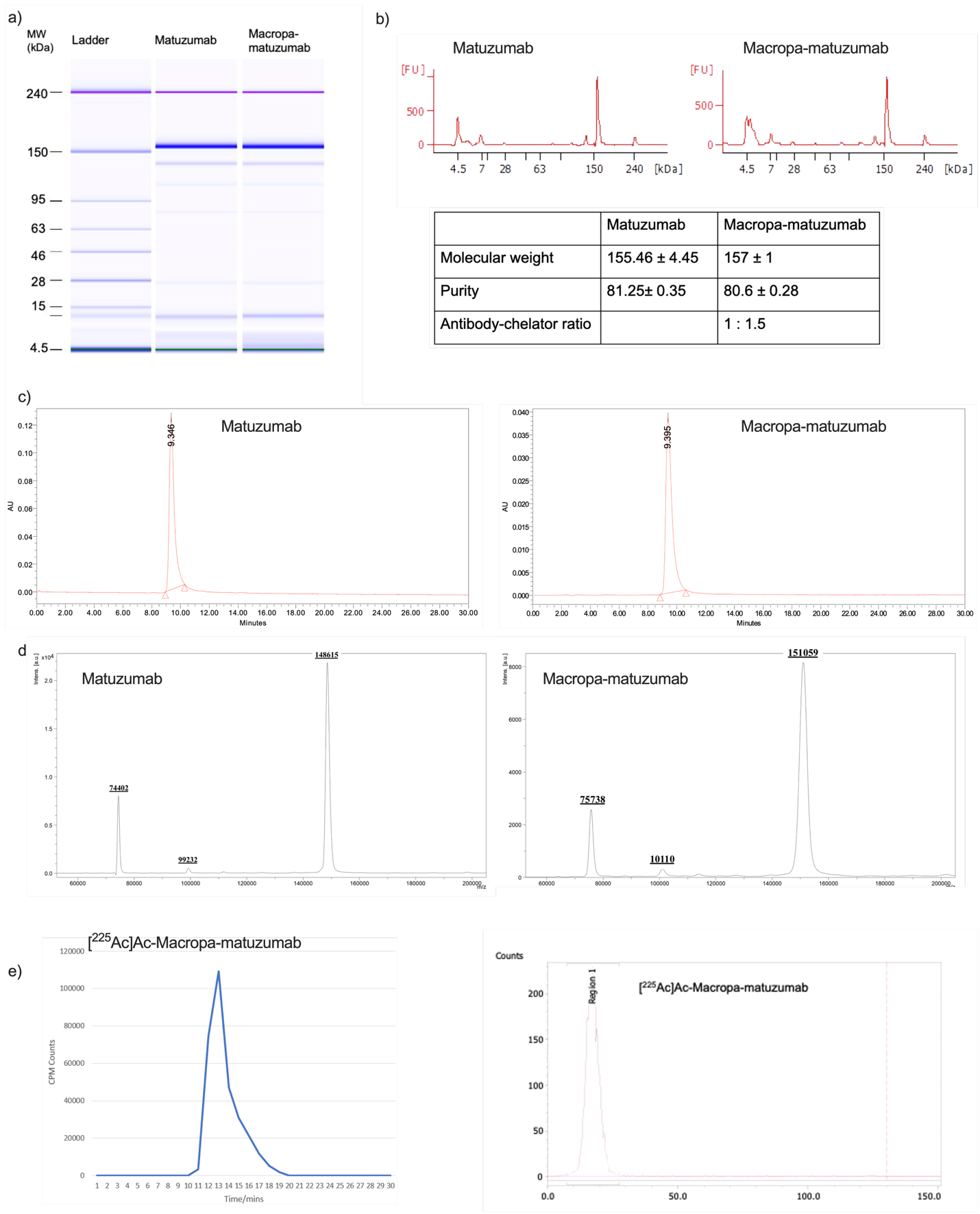


Figure S1: QC of antibodies and radioimmunoconjugate. A) Bioanalyzer gel and B) Electropherograms of matuzumab and Macropa-matuzumab. Molecular weights are in kDa, while purity is in percentage (%). C) Size-exclusion HPLC (SEC-HPLC) and D) MALDI-TOF mass spectra of matuzumab and Macropa-matuzumab. E) radio- SEC-HPLC (left) and iTLC (right) of [^225^Ac]Ac-Macropa-matuzumab.

Bioanalyzer gels were obtained by electronic electrophoresis, and the size and relative peak area of the constructs were calculated using Agilent 2100 Expert software. SEC-HPLC was performed using Waters 2796 Bioseparation modules, Water 2487 Absorbance Detector, and XBridge® BEH 200A SEC 3.5 μm 7.8 × 150 mm column. The UV detector was set at 254 and 280 nm with PBS as solvent and a flow rate of 0.45 mL/min. For iTLC, 1-5 µL was spotted on silica gel-impregnated paper (iTLC-SG) and developed using mobile phase of 50 mM sodium citrate buffer (pH 5.2).


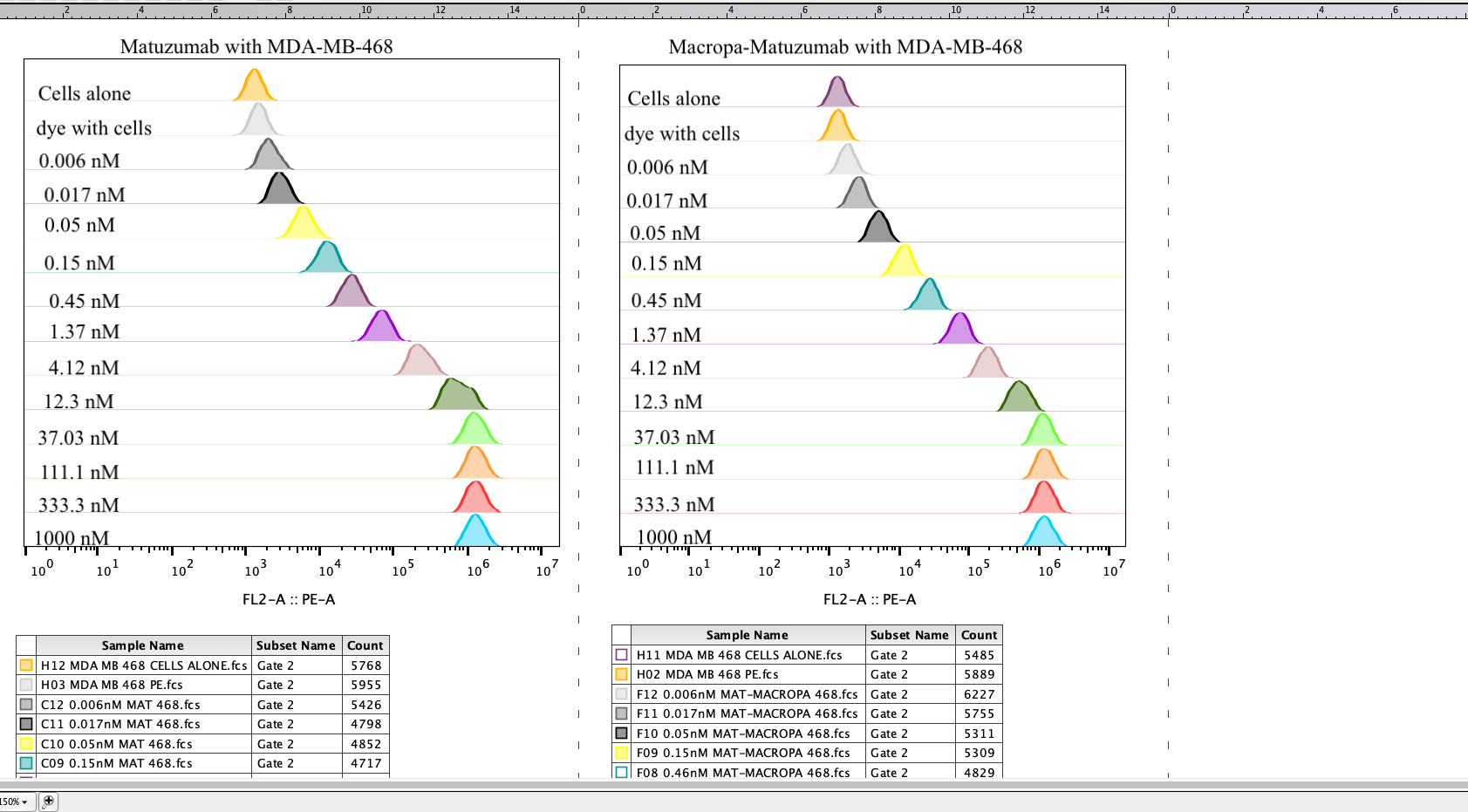

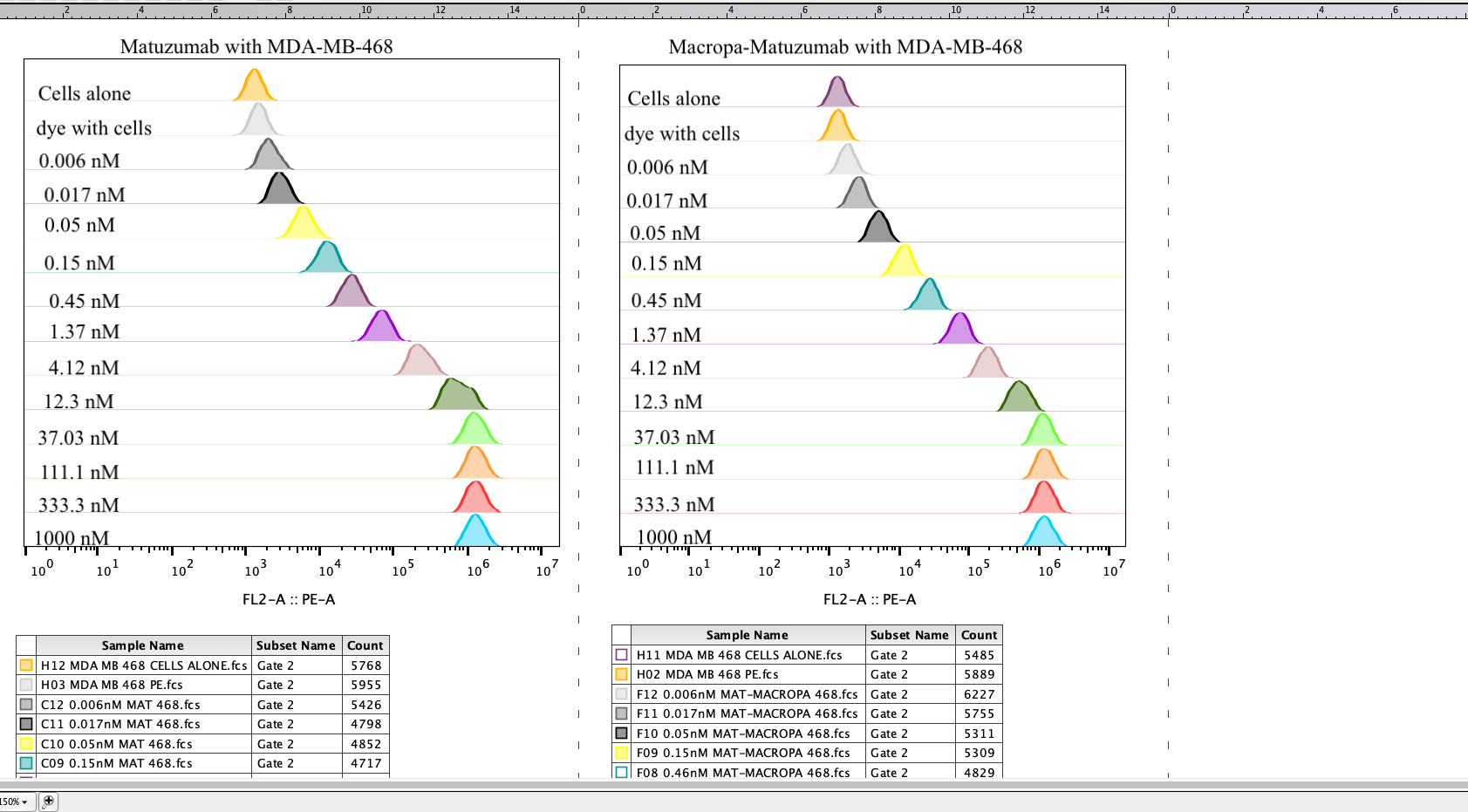


Macropa-matuzumab with MDA-MB-468


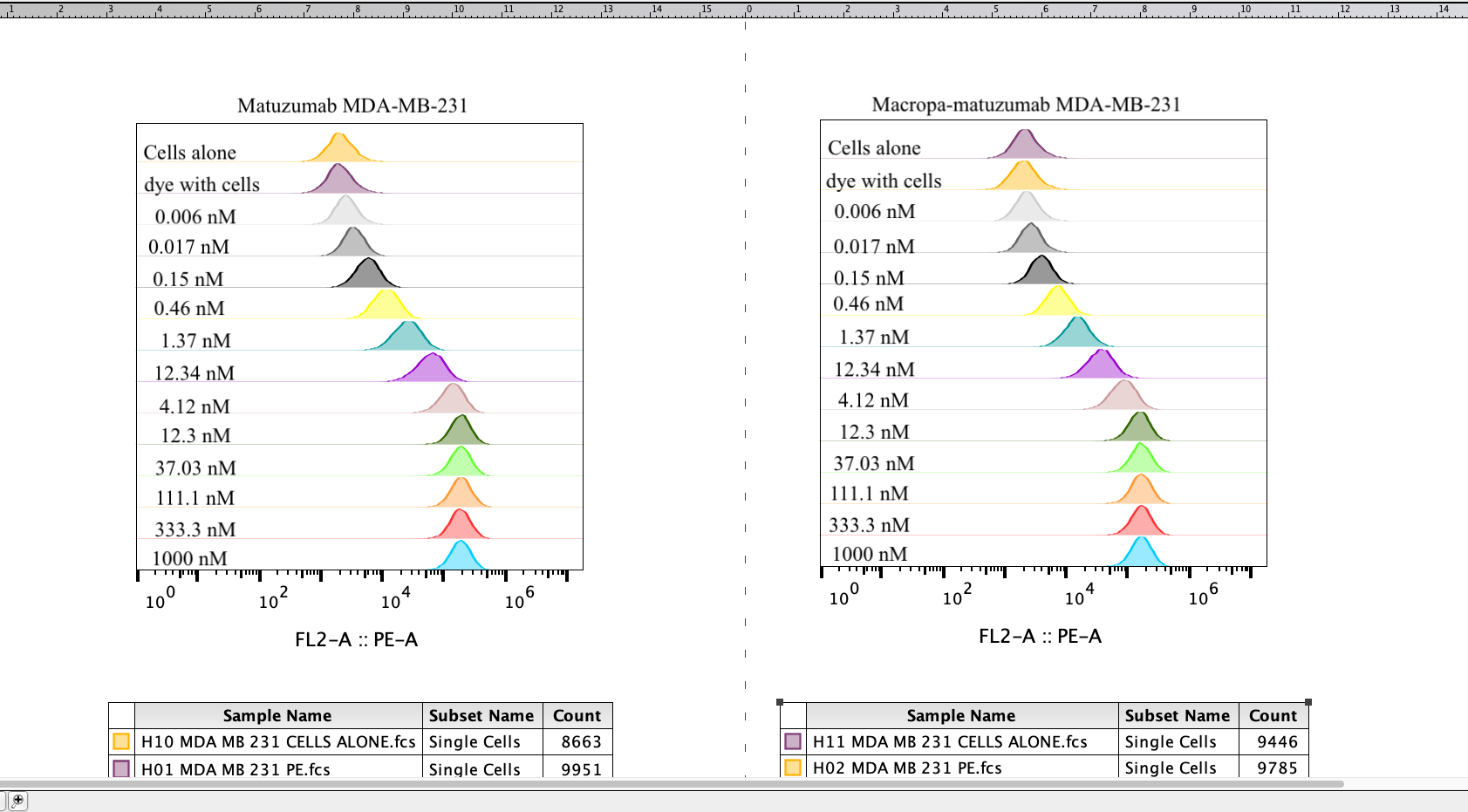

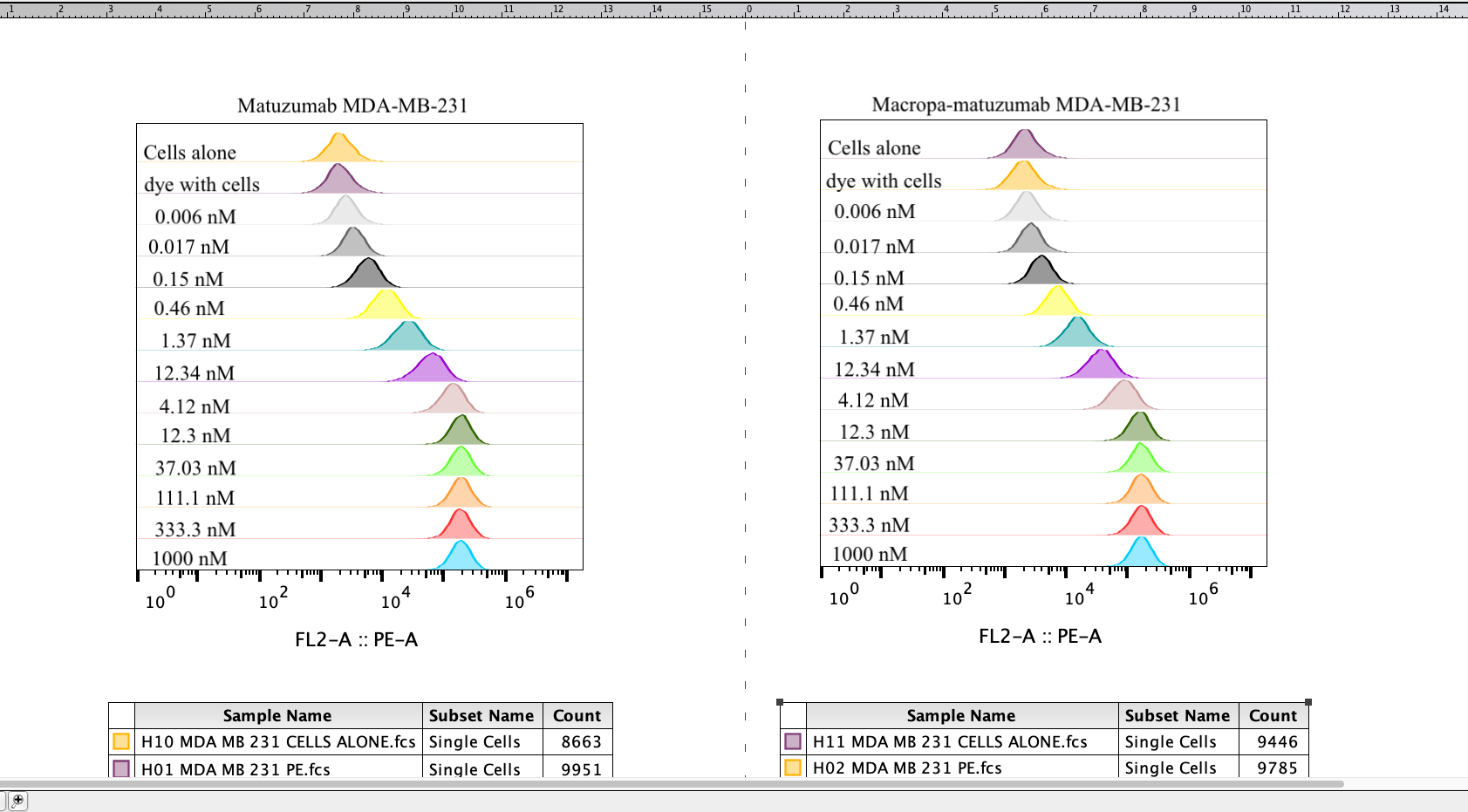


Figure S2: Binding of matuzumab and Macropa-matuzumab in EGFR-positive cells. In vitro flow cytometry histograms of matuzumab and Macropa-matuzumab in MDA-MB-468 and MDA-MB-231 with high and medium EGFR density, respectively, using different concentrations of the antibodies. Matuzumab and Macropa-matuzumab were left to bind to MDA-MB-468 and MDA-MB-231 cells for 30 mins at 4 °C, after which cells were washed. Goat anti-Human IgG PE-conjugated secondary antibody (eBioscience, cat. #12-4998-82) was added and left to incubate for 30 mins at 4 °C. Cells were further washed and resuspended in 1X DPBS. Flow cytometry was performed using the BD Accuri^TM^ C6 Plus flow cytometer (BD Bioscience, Franklin Lakes, NJ). The data were analyzed using FlowJo v10 (BD Biosciences, Ashland OR).


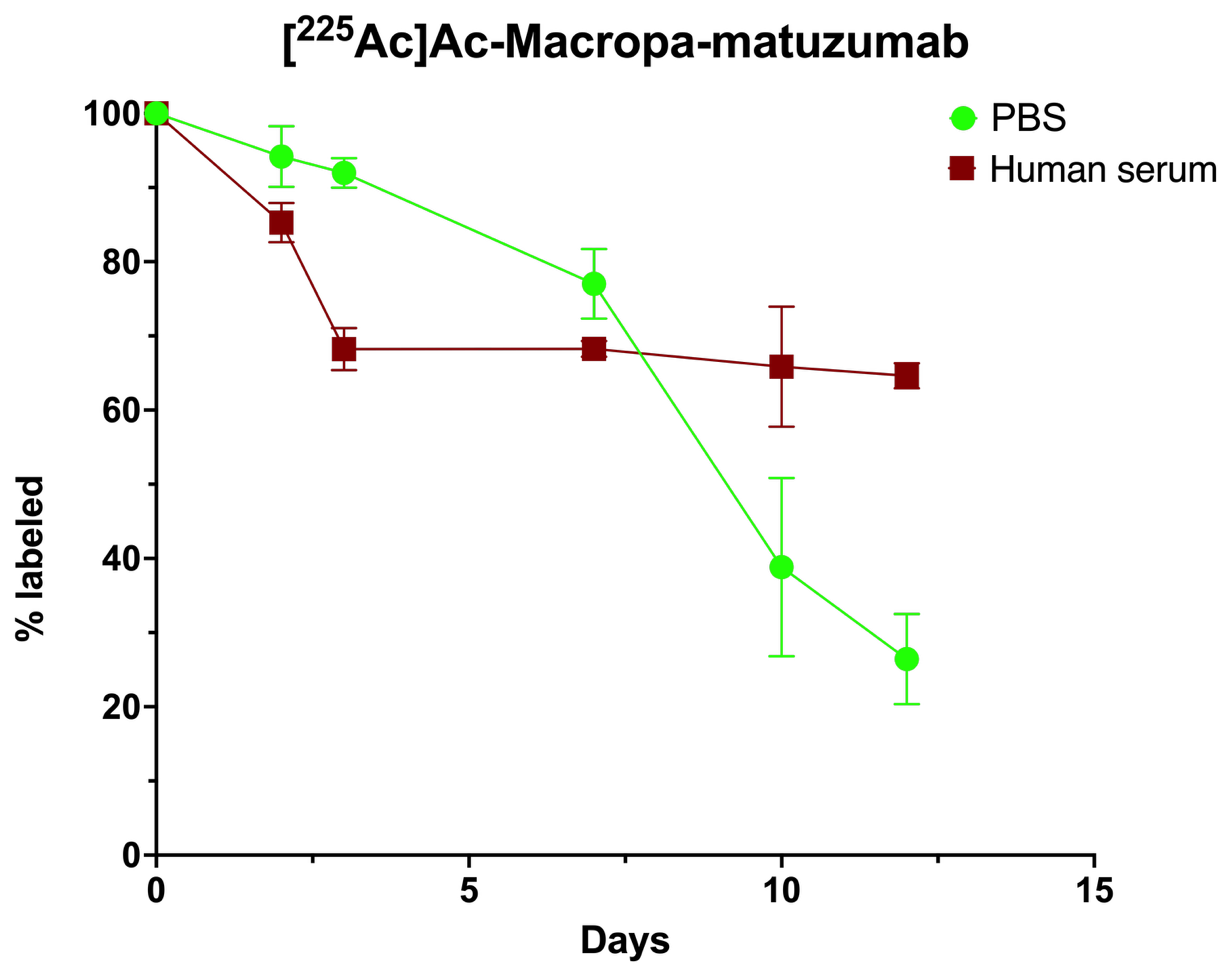


Figure S3: Stability of [^225^Ac]Ac-Macropa-matuzumab in PBS and human serum over a period of 12 days. The radioimmunoconjugate was incubated at 37 °C in PBS and human serum, and the % radiolabeled antibody was measured on days 0, 2, 3, 7, 10, and 12 to assess its stability.


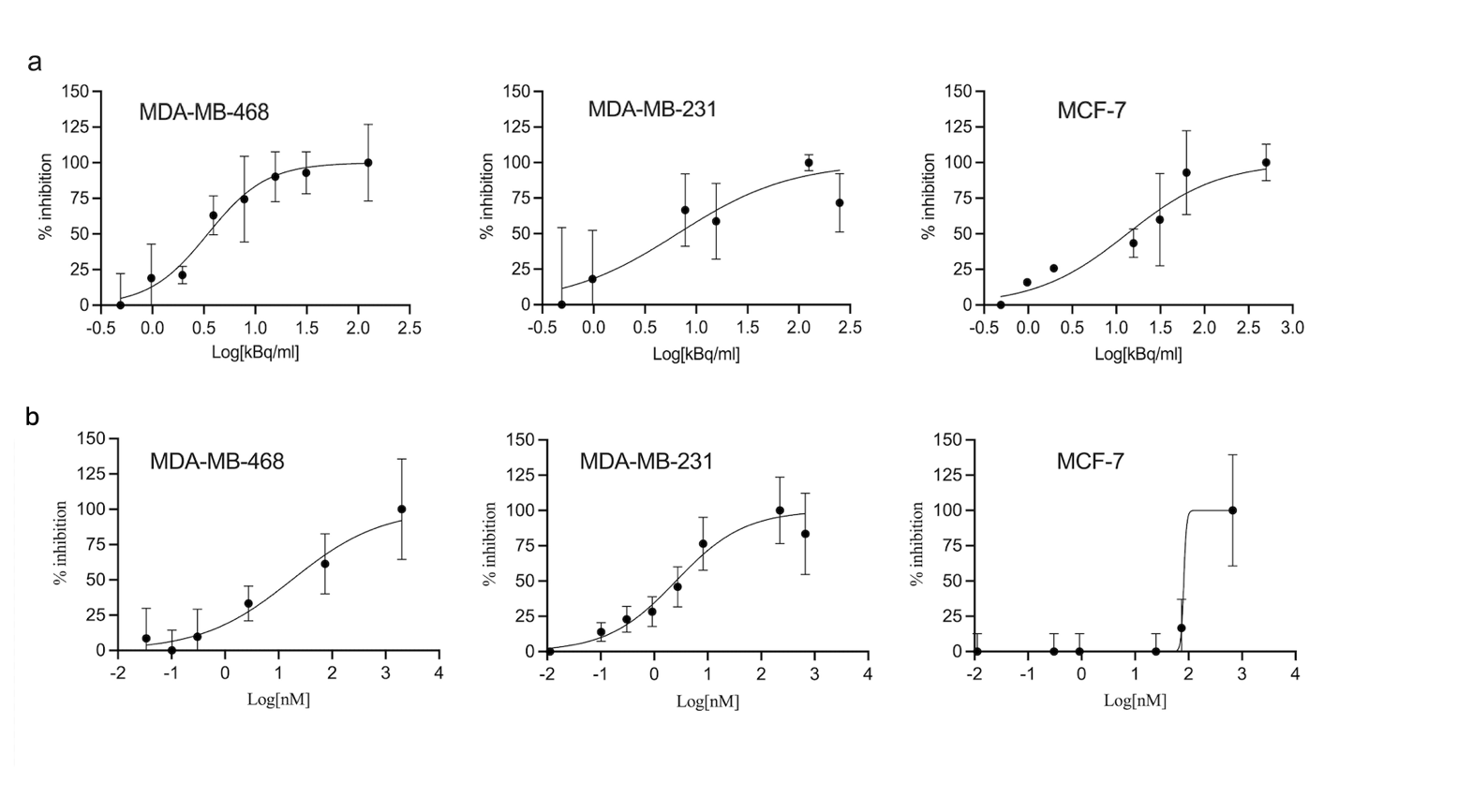


Figure S4**:** *In vitro* cytotoxicity in monolayer cells (2D). Cytotoxicity of a) [^225^Ac]Ac-Macropa-matuzumab and b) matuzumab in cell lines with different levels of EGFR expression (MDA-MB-468, high; MDA-MB-231, medium; MCF-7, low expression). Incucyte^®^ Cytotox Red reagent was used to stain dead cells after treatment with different concentrations of [^225^Ac]Ac-Macropa-matuzumab (250 - 0.48 KBq/mL) or matuzumab (2000 nM - 0.033 nM). Images were captured with an IncuCyte S3 Live cell imaging system, and the relative fluorescent values generated were used to calculate the IC_50_ values using GraphPad Prism 10.


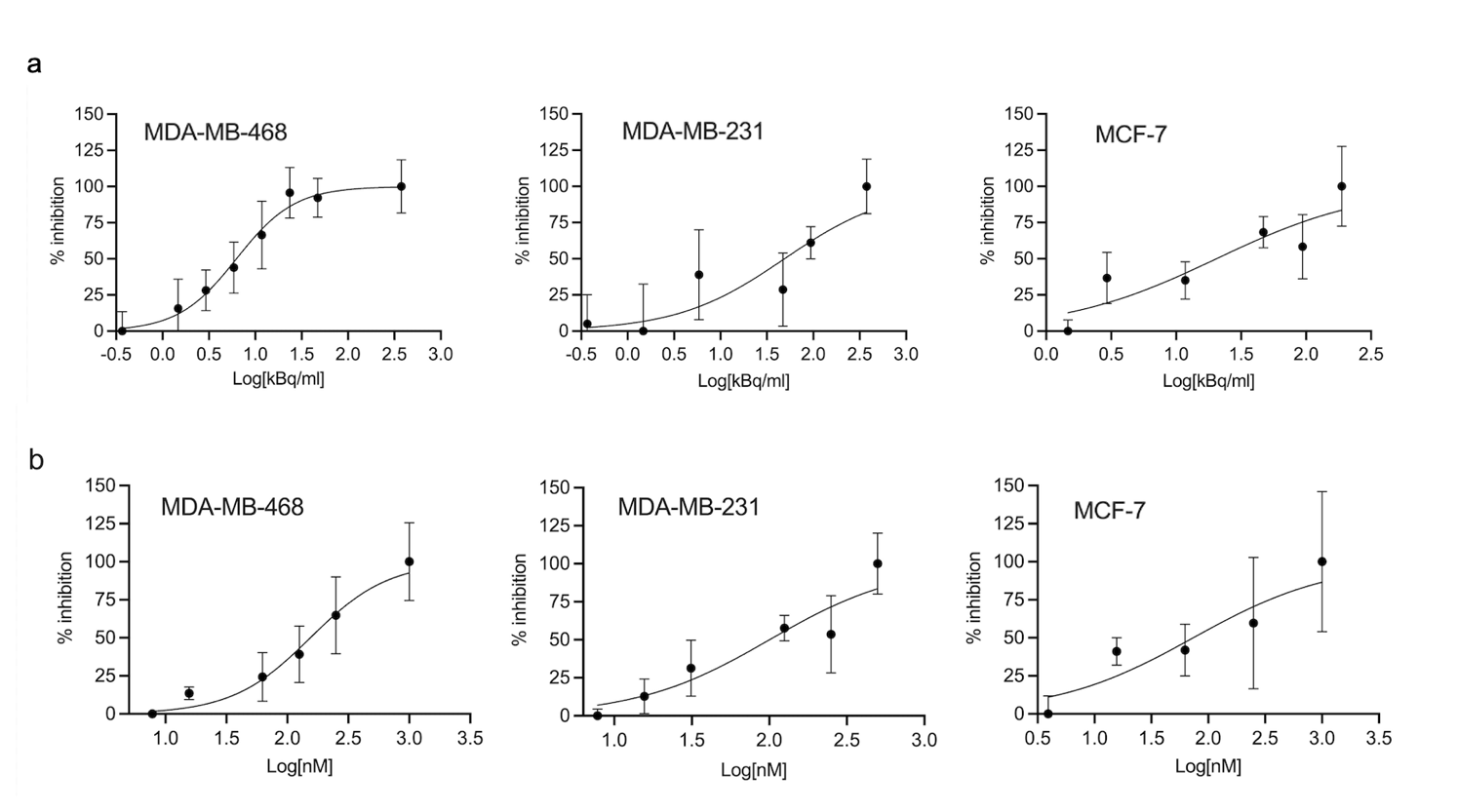


Figure S5: *In vitro* cytotoxicity in spheroids (3D). Cytotoxicity of a) [^225^Ac]Ac-Macropa-matuzumab and b) matuzumab in spheroids with different levels of EGFR expression (MDA-MB-468, high; MDA-MB-231, medium; MCF-7, low expression). Spheroids were established by culturing cells in collagen 1 containing complete growth media. Cells were treated with different concentrations of [^225^Ac]Ac-Macropa-matuzumab (250 - 0.48 KBq/mL) or matuzumab (2000 nM – 3.9 nM) in Incucyte^®^ Cytotox Red reagent containing media. Fluorescent images were collected using an IncuCyte S3, and fluorescence outputs were analyzed in GraphPad Prism 10 to derive IC₅₀ values.

**Table 1**: Toxicity of [^225^Ac]Ac-Macropa-matuzumab in healthy female mice (n = 4 per group). Mice were injected with 2 doses of 13 kBq [^225^Ac]Ac-Macropa-matuzumab and blood was collected for analysis 14 days after the second dose.

|  | Units | Saline | [^225^Ac]Ac-Macropa-matuzumab | p value |
| --- | --- | --- | --- | --- |
| WBC | x10^9^/L | 4.1 ± 0.8 | 0.5 ± 0.4 | * |
| RBC | x10^12/L | 9.5 ± 0.2 | 5.4 ± 1.3 | ns |
| Hgb | g/L | 146 ± 3.6 | 85.0 ± 23.0 | ns |
| Hct | L/L | 0.45 ± 0.02 | 0.24 ± 0.09 | ns |
| MCV | fL | 48.1 ± 0.4 | 44.4 ± 1.6 | ns |
| MCH | pg | 15.4 ± 0.2 | 15.7 ± 0.5 | ns |
| MCHC | g/L | 319.5 ± 2.0 | 353.0 ± 1.0 | *** |
| RDW | % | 13.0 ± 0.2 | 13.8 ± 0.42 | ns |
| Platelets | x10^9/L | 70.3 ± 11.84 | 9.5 ± 0.5 | * |
| Urea | mmol/L | 7.3 ± 0.2 | 3.5 ± 0.5 | * |
| Creatinine | $\mu$mol/L | 12.0 ± 2.3 | 21.3 ± 2.2 | * |
| Glucose | mmol/L | 10.9 ± 0.7 | 10.2 ± 0.2 | ns |
| Alk Phos | mmol/L | 125.3 ± 6.6 | 138.0 ± 4.4 | * |
| ALT | U/L | 26.3 ± 2.8 | 216.0 ± 15.9 | ** |
| GLDH | U/L | 8.3 ± 0.7 | 80.7 ± 13.8 | * |
| AST | U/L | 380.4 ± 225.3 | 1131.0 ± 118.3 | ns |
| Total protein | g/L | 46.7 ± 1.9 | 44.3 ± 1.8 | ns |

ns = nonsignificant ( p-value > 0.05), * = p-value < 0.05, ** = p-value < 0.001, *** = p-value < 0.0001. White blood cells (WBC), red blood cells (RBC), hemoglobin (Hgb), hematocrit (Hct), mean corpuscular volume (MCV), mean corpuscular hemoglobin (MCH), mean corpuscular hemoglobin concentration (MCHC), red cell distribution width (RDW), alkaline phosphatase (ALP), alanine transaminase (ALT), glutamate dehydrogenase (GLDH), aspartate aminotransferase (AST)

Table 2: Biodistribution of [^225^Ac]Ac-Macropa-matuzumab in healthy BALB/c mice (n = 4 per time point) at different time points. Mice were injected with 13 kBq of [^225^Ac]Ac-Macropa-matuzumab, sacrificed at different time points, and the carcasses were collected and measured using a gamma counter.

|  | Blood and tissue Uptake (%IA/g ± SEM) | | | | |
| --- | --- | --- | --- | --- | --- |
| Organs | 5 h | 24 h | 72 h | 120 h | 240 h |
| Bladder | 2.7 ± 0.2 | 6.2 ± 1.0 | 8.0 0.5 | 6.5 ± 1.1 | 1.2 ± 1.2 |
| Kidney | 10.9 ± 0.4 | 12.2 ± 1.6 | 9.1 ± 0.6 | 9.4 ± 0.5 | 8.5 ± 0.6 |
| Liver | 16.4 ± 0.7 | 10.9 ± 1.2 | 11.7 ± 0.6 | 11.7 ± 0,7 | 11.2 ± 1.2 |
| Pancreas | 2.6 ± 0.3 | 3.1 ± 0.5 | 2.8 ± 0.3 | 2.7 ± 0.6 | 1.5 ± 0.1 |
| Spleen | 23.1 ± 2.0 | 17.0 ± 2.0 | 17.5 ± 0.2 | 21.8 ± 1.3 | 27.6 ± 3.6 |
| Lungs | 13.2 ± 1.8 | 9.2 ± 1.1 | 8.2 ± 1.2 | 8.8 ± 0.3 | 7.5 ± 0.5 |
| Heart | 8.8 ± 0.5 | 6.0 ± 0.5 | 5.4 ± 0.5 | 4.8 ± 0.4 | 3.4 ± 0.1 |
| Large intestine | 3.4 ± 0.3 | 2.2 ± 0.2 | 2.5 ± 0.2 | 3.1 ± 0.4 | 2.3 ± 0.1 |
| Smal Intestine | 5.0 ± 0.4 | 3.7 ± 0.3 | 3.0 ± 0.9 | 3.6 ± 0.4 | 3.3 ± 0.1 |
| Stomach | 2.2 ± 0.4 | 1.8 ± 0.2 | 2.4 ± 0.4 | 1.9 ± 0.2 | 1.5 ± 0.1 |
| Skull | 4.2 ± 0.3 | 4.4 ± 0.5 | 4.2 ± 0.2 | 4.2 ± 0.3 | 3.5 ± 0.3 |
| Brain | 0.8 ± 0.0 | 0.6 ± 0.1 | 0.5 ± 0.1 | 0.3 ± 0.1 | 0.2 ± 0.0 |
| Limbs | 2.9 ± 0.0 | 3.3 ± 0.4 | 3.5 ± 0.2 | 3.5 ± 0.2 | 2.6 ± 0.3 |
| Spine | 2.9 ± 0.3 | 2.9 ± 0.5 | 3.0 ± 0.2 | 3.2 ± 0.3 | 2.7 ± 0.2 |
| Blood | 37.6 ± 1.2 | 22.2 ± 1.3 | 19.7 ± 1.2 | 20.1 ± 1.0 | 13.7 ± 2.1 |
| Bone | 4.1 ± 0.2 | 3.7 ± 0.4 | 3.5 ± 0.1 | 3.2 ± 0.1 | 2.4 ± 0.4 |
| Muscles | 1.8 ± 0.1 | 2.5 ± 0.7 | 3.5 ± 0.3 | 3.0 ± 0.5 | 1.6 ± 0.3 |
| Tail | 8.7 ± 2.1 | 8.3 ± 3.3 | 4.6 ± 0.3 | 5.2 ± 0.3 | 3.7 ± 0.1 |
| skin | 3.9 ± 0.3 | 6.0 ± 0.8 | 6.4 ± 0.2 | 5.7 ± 0.3 | 4.4 ± 0.3 |
| Uterus | 4.9 ± 0.2 | 4.3 ± 0.7 | 4.9 ± 0.3 | 4.7 ± 0.3 | 2.4 ± 1.1 |
| Ovaries | 7.6 ± 2.8 | 5.2 ± 0.7 | 6.3 ± 0.6 | 7.2 ± 1.0 | 5.8 ± 1.3 |
| Adrenals | 6.9 ± 1.2 | 6.1 ± 0.7 | 3.9 ± 1.4 | 1.6 ± 0.6 | 0.2 ± 0.2 |
| Thymus | 5.1 ± 1.6 | 6.2 ± 1.8 | 5.2 ± 0.7 | 4.6 ± 1.3 | 1.7 ± 0.6 |
| Eyes | 1.9 ± 0.2 | 1.8 ± 0.3 | 2.2 ± 0.3 | 1.3 ± 0.3 | 0.6 ± 0.2 |
| Gallbladder | 5.1 ± 2.5 | 1.8 ± 1.4 | 3.2 ± 1.4 | 0.1 ± 0.1 | 1.1 ± 1.1 |
| Thyroid | 5.2 ± 1.5 | 4.6 ± 0.3 | 5.2 ± 1.0 | 4.6 ± 1.0 | 1.0 ± 0.3 |

Table 3: Biodistribution of [^225^Ac]Ac-Macropa-matuzumab in tumor-bearing athymic nude BALB/c mice, value ± SEM, n=4 at 24 h, and n=3 at 120 h and 240 h

|  | Blood and tissue Uptake (%IA/g ± SEM) | | |
| --- | --- | --- | --- |
| Organs | 24 h | 120 h | 240 h |
| Bladder | 3.4 ± 0.8 | 1.5 ± 1.5 | 0.0 ± 0.0 |
| Kidney | 9.1 ± 1.0 | 3.6 ± 0.8 | 1.9 ± 1.1 |
| Liver | 13.5 ± 0.9 | 7.5 ± 0.4 | 5.5 ± 0.6 |
| Pancreas | 1.9 ± 0.2 | 0.8 ± 0.4 | 0.2 ± 0.2 |
| Spleen | 14.4 ± 2.6 | 6.9 ± 2.0 | 3.3 ± 0.4 |
| Lungs | 6.5 ± 0.4 | 2.5 ± 0.6 | 0.7 ± 0.6 |
| Heart | 4.0 ± 0.2 | 1.0 ± 0.3 | 0.4 ± 0.4 |
| Large intestine | 1.6 ± 0.3 | 0.6 ± 0.1 | 0.3 ± 0.1 |
| Smal Intestine | 2.7 ± 0.7 | 1.0 ± 0.2 | 0.4 ± 0.2 |
| Stomach | 1.0 ± 0.2 | 0.4 ± 0.1 | 0.1 ± 0.1 |
| Brain | 0.3 ± 0.1 | 0.1 ± 0.1 | 0.0 ± 0.0 |
| Blood | 16.7 ± 1.4 | 5.1 ± 1.4 | 2.2 ± 1.7 |
| Bone | 3.0 ± 0.2 | 1.2 ± 0.2 | 0.7 ± 0.4 |
| skin | 7.2 ± 0.3 | 2.9 ± 0.2 | 1.5 ± 0.8 |
| Tumor-468 | 36.7 ± 3.9 | 34.6 ± 5.7 | 26.9 ± 13.4 |
| Tumor-231 | 28.5 ± 2.0 | 36.0 ± 4.9 | 15.4 ± 8.4 |
